# Accurate Detection and Analysis of Pore Defects in Laser Powder Bed Fusion WE43 Magnesium Alloys

**DOI:** 10.3390/mi15070909

**Published:** 2024-07-12

**Authors:** Zhengxing Men, Liang Wang, Xi Gao, Wen Chen, Chen Ji, Ziche Li, Kun Li

**Affiliations:** 1 Aviation Equipment Manufacturing Industry College, Chengdu Aeronautic Polytechnic, Chengdu 610100, China; 2 College of Mechanical and Vehicle Engineering, Chongqing University, Chongqing 400044, China; 3 Chongqing Key Laboratory of Metal Additive Manufacturing (3D Printing), Chongqing University, Chongqing 400044, China

**Keywords:** laser powder bed fusion, porosity defects, sphericity, WE43 magnesium alloy

## Abstract

To explore the size, morphology, and distribution patterns of internal pore defects in WE43 magnesium alloy formed by laser powder bed fusion (LPBF), as well as their impact on its mechanical properties, computer tomography (CT), metallographic microscopy, and scanning electron microscopy were used to observe the material’s microstructure and the morphology of tensile test fractures. The study revealed that a large number of randomly distributed non-circular pore defects exist internally in the LPBF-formed WE43 magnesium alloy, with a defect volume fraction of 0.16%. Approximately 80% of the defects had equivalent diameters concentrated in the range of 10∼40 μm, and 56.2% of the defects had sphericity values between 0.65∼0.7 μm, with the maximum defect equivalent diameter being 122 μm. There were a few spherical pores around 20 μm in diameter in the specimens, and unfused powder particles were found in pore defects near the edges of the parts. Under the test conditions, the fusion pool structure of LPBF-formed WE43 magnesium alloy resembled a semi-elliptical shape with a height of around 66 μm, capable of fusion three layers of powder material in a single pass. Columnar grains formed at the edge of individual fusion pools, while the central area exhibited equiaxed grains. The “scale-like pattern” formed by overlapping fusion pool structures resulted in the microstructure of LPBF-formed WE43 magnesium alloy mainly consisting of fine equiaxed grains with a size of 2.5 μm and columnar grains distributed in a band-like manner.

## 1. Introduction

Laser powder bed fusion (LPBF) technology is currently the most commonly used method for direct additive manufacturing of metal materials, and is widely applied used in rapid manufacturing of complex parts.The inevitable formation of void defects during LPBF processing compromises the continuity of the metal, leading to stress concentration and crack initiation [1,2,3,4]. This is a primary cause for the mechanical instability, low ductility, and poor long-term mechanical performance of materials. The LPBF fabrication of magnesium alloy materials represents a novel forming method in the field of magnesium alloy manufacturing, currently in its early development stage [5,6].

The origins of void defects in LPBF can be attributed to three main factors: (1) Low laser energy density: During LPBF, insufficient laser energy can result in areas of incomplete fusion and inadequate solidification, leading to irregularly shaped voids. The formation of such defects is closely related to process parameters such as insufficient laser power, high build speeds, or excessive powder layer thickness. In extreme cases, unfusioned powder particles can be found within these void defects. It is hard to completely eliminate unfusioned voids, because the rapid scanning of the laser in multiple directions during LPBF, leading to uneven surface, which will make it difficult to control the powder thickness [7,8]. (2) High laser energy density: Excessively high laser energy can cause rapid evaporation of metal, which will generate strong recoil pressure that pushes the surrounding molten metal downward, forming deep and narrow keyhole-shaped defects. Complete keyholes are typically found only on the top layer of the part; internally, they can transform into multiple adjacent voids during subsequent refusion cycles, making them indistinguishable from unfusioned voids. High laser energy density can also lead to defects such as turbulence and spattering, which accumulate layer by layer to form void defects [9,10,11]. And (3) Gas pores: Gas pore defects typically exhibit smooth surfaces and are spherical or near-spherical in shape. Gas pores may arise from external factors, such as inert gas blown into the fusion pool that fails to escape in time, or from high gas content and low packing density of the powder material [12,13,14].

With the widespread application of LPBF technology, significant progress has been made in the research of non-destructive testing methods for LPBF-formed components. Rees [4] used high-resolution synchrotron X-ray computed tomography (sCT) to measure the volume fraction of defects, such as pores and microcracks, in the completed LPBF samples. Captured crack propagation, pore evolution, wetting behavior, and construction height exceeding 1.0 ms at a rate of approximately 110 mm/s. Zhou [15] found, through X-ray phase analysis, that TC4 underwent a phase transition. Calta [16] used in situ X-ray measurements of laser powder bed fusion (LPBF) additive manufacturing processes to generate unique data for model validation and improved process understanding.

This study focuses on the WE43 magnesium alloy, and the size, morphology, and distribution characteristics of void defects in magnesium alloy materials during LPBF forming were analyzed by the CT non-destructive testing methods and metallographic observation. This research not only helps to optimize process parameters for LPBF fabrication of magnesium alloy components, but also provides strong theoretical support and practical guidance for subsequent process planning.

## 2. Experimental

**Figure 1:**
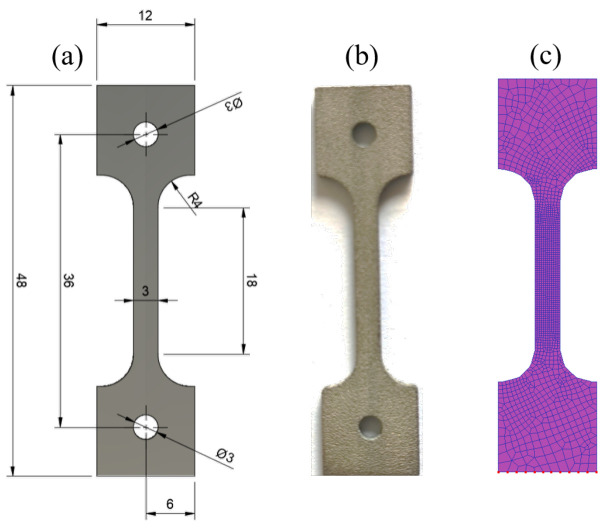
WE43 magnesium alloy tensile specimen. (**a**) Size of WE43 tensile sample formed by LPED. (**b**) Sample physical object. (**c**) Mesh partitioning for finite element analysis.

The chemical composition of WE43 magnesium alloy powder material is shown in Table 1. The dimensions of the tensile specimen is illustrated in Figure 1a. The tensile specimen obtained using the LPBF technology is depicted in Figure 1b, with its main forming parameters detailed in Table 2 [17,18]. The formed specimen was separated from the substrate using wire cutting. Figure 1c shows the two-dimensional finite element model of the tensile specimen, utilizing tetrahedral meshing with a total of 1500 elements.

The standard gauge section of the specimen was subjected to CT scanning using X-ray 3D microscopy equipment (nano voxel 2000, Cray, Japan) under the following conditions: voltage of 80 kV, current of 100 μA, resolution of 11.50 μm, and a testing duration of 1.5 h. Tensile testing at room temperature was performed using an E45.350 (MTS, Eden Prairie, MN, USA) servo-hydraulic testing machine at a strain rate of 0.0015 mm/min. The fracture surface was examined using a S-3400 scanning, Hitachi, Japan electron microscope. The microstructure of the WE43 magnesium alloy was observed using a DM2700M metallurgical microscope, Zeiss, Jena, Germany, with a 1% nitric acid alcohol etchant applied for 2 s during sample preparation.

## 3. Experimental Results Analysis

### 3.1. CT Inspection and Analysis

**Figure 2:**
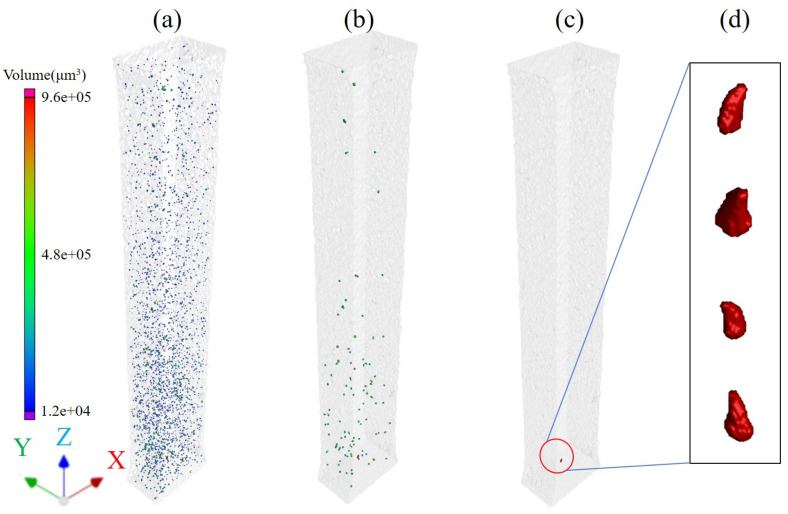
CT inspection results of LPBF-formed WE43 magnesium alloy. (**a**) Pore defects with an equivalent diameter of 10 μm and larger. (**b**) Pore defects with an equivalent diameter in the range of 77 μm to 122 μm. (**c**) Pore defects with an equivalent diameter of 122.235 μm. Its sphericity is measured at 0.6, resembling a horn-like morphology in (**d**).

Figure 2 illustrates the distribution of void defects within the gauge Section (3 mm × 2 mm × 18 mm) of the LPBF formed WE43 magnesium alloy tensile specimen. As shown in Figure 2a, numerous void defects with equivalent diameters of 10 μm and larger are present within the gauge section of the specimen, exhibiting a random distribution pattern. There are no cracks, inclusions and other defects within this area. The largest volume defect within the specimen is depicted in Figure 2b, with an equivalent diameter ranging from 77 μm to 122 μm. The largest void defect in the specimen is located at the bottom region of Figure 2c, with an equivalent diameter of 122.235 μm and a surface area of 78,537.34 μm^2^. Its sphericity is measured at 0.6, resembling a horn-like morphology in Figure 2d.

**Figure 3:**
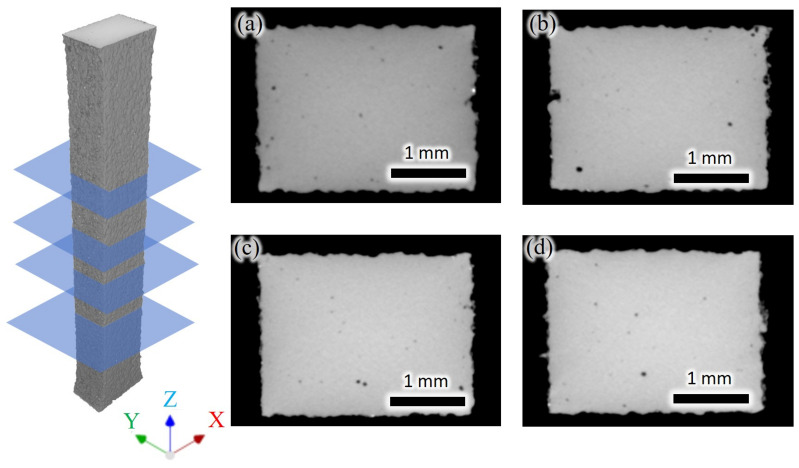
XY Cross-sectional CT Inspection Results of Tensile Specimen. (**a**) 2.37 mm from the bottom surface. (**b**) 9.54 mm from the bottom surface. (**c**) 15.43 mm from the bottom surface. (**d**) 19.14 mm from the bottom surface.

**Figure 4:**
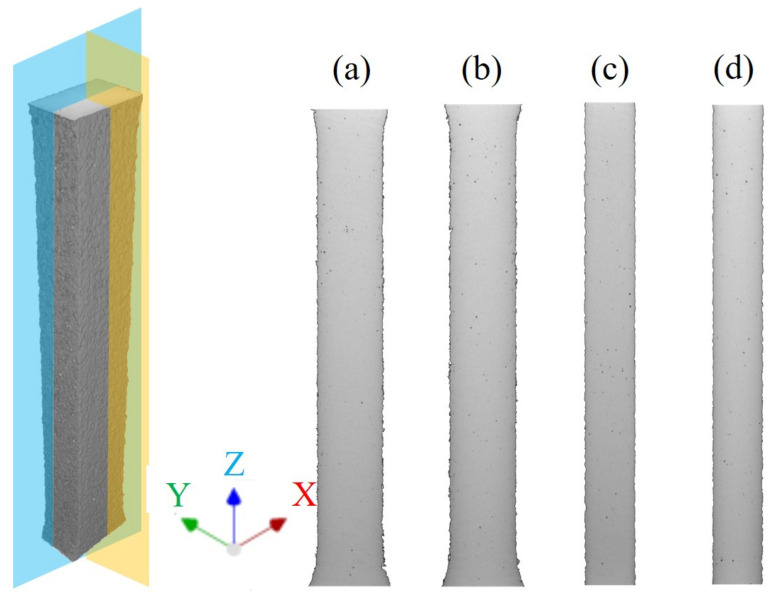
ZX and YZ cross-sectional CT inspection results of tensile specimen. (**a**) Parallel to the ZX plane and 0.58 mm away from the side. (**b**) Parallel to the ZX plane and 1.58 mm away from the side. (**c**) Parallel to the ZY plane and 1.12 mm away from the side. (**d**) Parallel to the ZY plane and 2.44 mm away from the side.

From the figure, it can be observed that the defects within the XY plane of the specimen are primarily categorized into internal defects and surface defects. Internal defects within the XY cross-sections appear as mostly circular voids, distributed randomly. The XY cross-section of the specimen’s surface is relatively rough, and basically wavy, with the presence of closed or semi-closed pore-like defects in Figure 3. The Z-direction CT scan results are depicted in ZX as shown in Figure 4a,b and YZ Figure 4c,d cross-sections.

**Figure 5:**
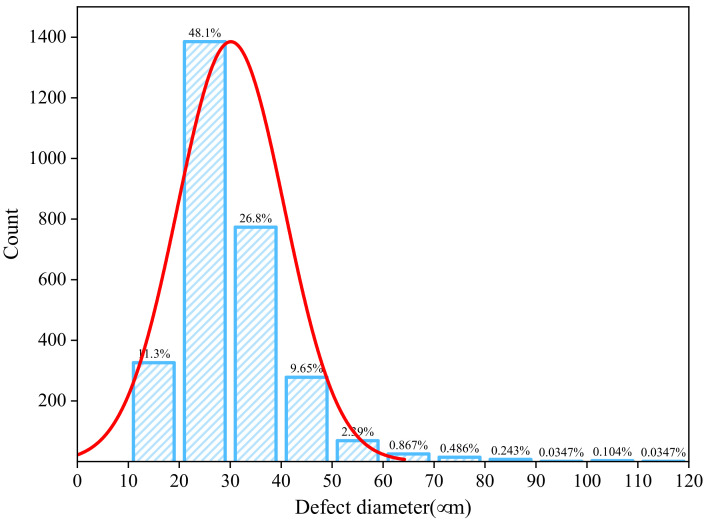
Distribution and normal distribution curve of defect sizes in WE43 magnesium alloy.

Within the entire gauge section of the specimen, a total of 2883 defects were identified, with a defect volume percentage of 0.16% and an average size of 30 μm. As depicted in Figure 5, approximately 45% of the defects have diameters concentrated in the range of 20∼30 μm, and about 80% of the defects have diameters within the range of 10∼40 μm. There are only 12 defects which have diameters larger than 80 μm.

**Figure 6:**
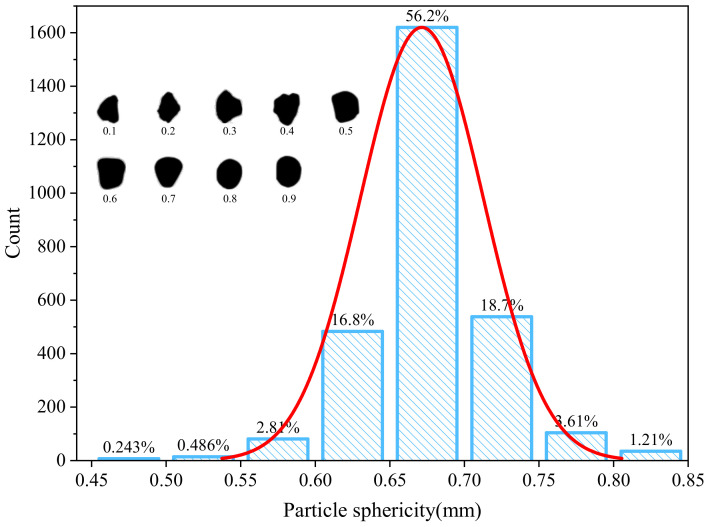
Sphericity and normal distribution curve distribution of defects in WE43 magnesium alloy.

Sphericity (S) is used to describe the three-dimensional shape of particles, and it is defined as the ratio between the surface area of a sphere (*A_sphere_*) with the same volume as the defect and the actual surface area of the defect (*A_defect_*), as shown in Equation (1). The value of S ranges from 0 to 1, where a value of 1 indicates perfect sphericity (a sphere), and lower values correspond to more elongated or irregular shapes, such as plate-like or columnar particles. Different sphericity values correspond to different defect morphologies, as illustrated in Figure 6. A higher S value indicates a defect shape closer to a perfect circle. According to Figure 6, 56.2% of the defects have S values in the range of 0.65∼0.7 mm, and only 1.21% of defects have S values exceeding 0.8, indicating fewer defects with circular shapes.

(1) $$Sphericity=\frac{A_{sphere}}{A_{defect}}$$

### 3.2. Low-Magnification Inspection and Analysis

**Figure 7:**
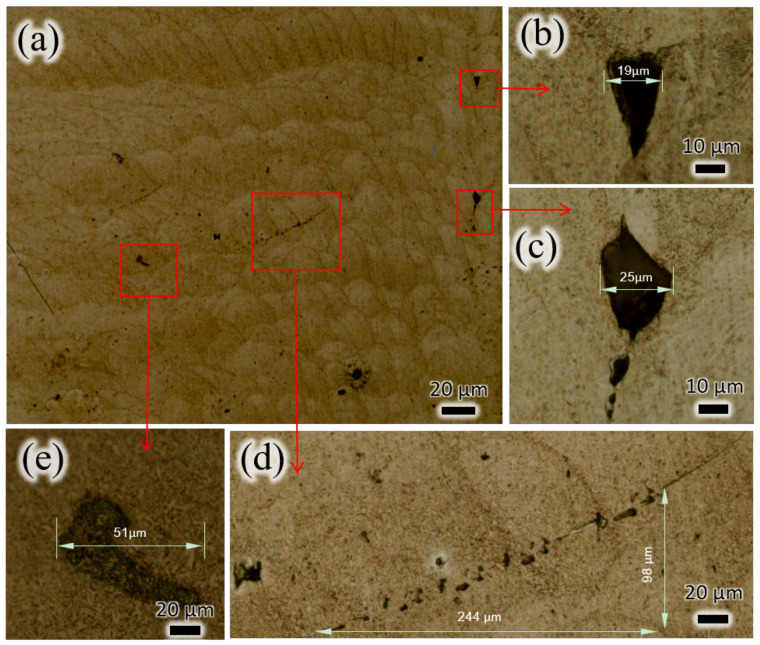
Appearance of WE43 prepared by LPBF. (**a**) Specific microstructural formations along the longitudinal cross-section of the part. (**b**,**e**)Isolated defects. (**c**,**d**) Continuous defects.

The LPBF deposition process is akin to multi-pass micro-zone laser welding. Each “scale” represents the trace left behind after rapid solidification of a single pass of the laser beam [19]. As the deposition process continues, there are local instances of multiple refusion and layer-by-layer stacking of the fusion pool, resulting in specific microstructural formations along the longitudinal cross-section of the part in Figure 7a. Based on CT inspection results, numerous irregularly shaped void defects with sizes below 50 μm were found internally within the part, with no cracks, inclusions, or other types of defects detected. These void defects can appear as isolated defects (as seen in Figure 7b,e) or as continuous defects (as in Figure 7c,d). In Figure 7d, a continuous line of small, hole-like defects is observed, crossing multiple “scales” with a total length of 244 μm. The occurrence of void defects is often at the edges of adjacent scales, with a few appearing within the scales themselves.

**Figure 8:**
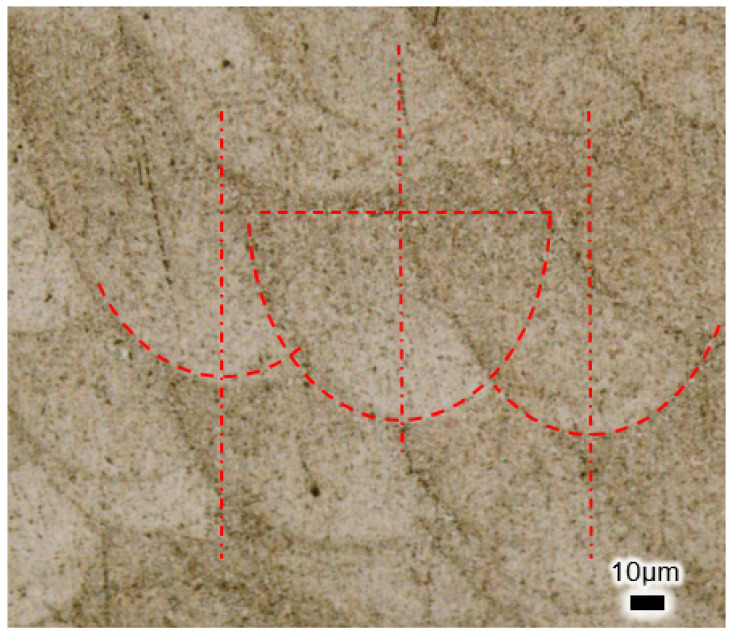
The outer surface Fusion pool structure of WE43 prepared by LPBF.

Figure 8 shows the formation of “scales” on the longitudinal cross-section of WE43 magnesium alloy under the specified experimental conditions, and the morphology of these scales is influenced by the LPBF process parameters. Using an ellipsoid model, the smallest width fusion pool depicted in Figure 8 is simplified for analysis. From the figure, it can be observed that three adjacent fusion pools conform to an ellipsoidal profile with dimensions of approximately 132 μm in length and 96 μm in width. The height of the fusion pool is about 66 μm, which corresponds to the thickness of three powder layers, indicating that during the LPBF process, each layer of WE43 magnesium alloy powder is refusioned at least three times.

### 3.3. High-Magnification Inspection and Analysis

**Figure 9:**
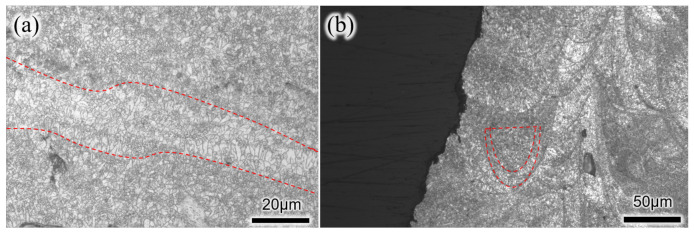
High magnification morphology of WE43 prepared by LPBF. (**a**) Cross sectional microstructure diagram of WE43 magnesium alloy formed by LPBF after etching. (**b**) Longitudinal cross-sectional microstructure diagram of the edge of WE43 magnesium alloy specimen formed by LPBF.

Under high-magnification microscopy, the microstructure of WE43 magnesium alloy produced by LPBF typically exhibits fine grains and a dense microstructure. This is attributed to the rapid fusion of powder particles by the high-energy-density laser beam in the LPBF process, followed by the formation of a dense metal structure through layer-by-layer deposition. Figure 9a shows a cross-sectional microstructure photo of LPBF-formed WE43 magnesium alloy after etching, revealing that the material is primarily composed of approximately 2.6 μm equiaxed grains with columnar grains distributed in a striped pattern, with a maximum grain aspect ratio of about 5.

During the LPBF process, with successive stacking of fusion pools, it becomes hard to achieve a fully refined structure. Figure 9b depicts the longitudinal section microstructure at the edge of an LPBF-formed WE43 magnesium alloy specimen, clearly showing that the presence of “scales” results in regular variations in grain size and morphology. Within individual fusion pools, the elongated grains formed at the pool edges are due to the rapid cooling rates at these edges, leading to significant thermal gradients affecting grain growth along the direction of heat flow. In contrast, the slower cooling rate in the central region of the fusion pool results in equiaxed grain formation.

### 3.4. Tensile Testing and Analysis

**Figure 10:**
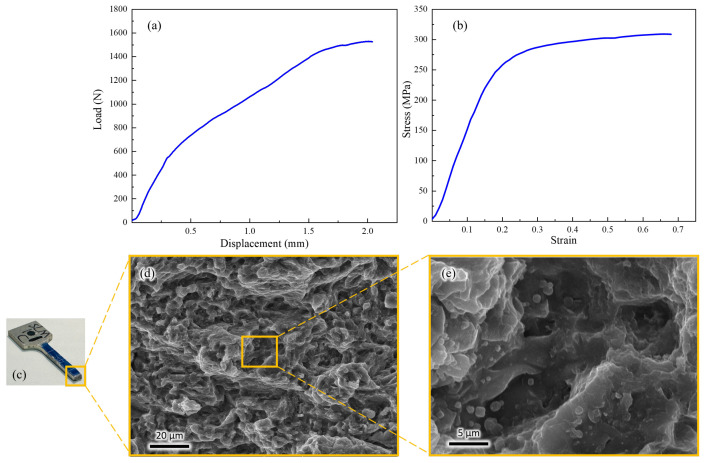
Room temperature tensile curve of LPBF-formed WE43. (**a**) The room temperature tensile curve of LPBF-formed WE43 magnesium alloy. (**b**) Stress–strain curve of LPBF formed D-WE43 magnesium alloy. (**c**) Neck contraction diagram of the fracture area. (**d**,**e**) Fracture surface of WE43 magnesium alloy.

The room temperature tensile curve of LPBF-formed WE43 magnesium alloy is shown in Figure 10a, with a maximum displacement of 2.04 mm and a maximum load of 1526.9 N, exhibiting no clear yield plateau. Figure 10b presents the rheological stress curve of LPBF-formed WE43 magnesium alloy, showing an ultimate tensile strength of 313 ± 5 MPa, a yield strength of 236 ± 12 MPa, and an elongation of 7.6 ± 0.5%. Macroscopically, necking phenomena are observed in the fracture region is showed in Figure 10c. Figure 10d,e display the fracture surface of WE43 magnesium alloy, exhibiting a typical ductile fracture morphology with numerous dimples that are uniformly sized and shaped, indicating the absence of significant defects.

## 4. Discussion

### 4.1. Porosity Formation Mechanism and Effects

In the LPBF process, the formation of pores is mainly due to the anomalous thermal history resulting from the rapid heating and cooling during the laser-metal interaction [20]. This produces a variety of pores, including air holes, incomplete fusion holes, and keyhole pores. Keyhole vents are particularly produced at higher laser energy densities and are caused by the trapping of gases by keyhole collapse due to strong evaporation. The presence of pores disrupts the continuity of the material and negatively affects the mechanical properties of the material. They become points of stress concentration, leading to fracture of the material at lower stresses.

### 4.2. Impact of the Size and Number of Pores

Porosity: Porosity is the ratio of the total volume of pores in the material to the total volume of the material. The higher the porosity, the lower the tensile strength of the material. According to the relevant research, when the porosity exceeds 5%, the effect on the strength of the material begins to become significant.

Pore size: The size of the pores is also critical to the strength of the material. Large pores are more likely to be points of stress concentration, causing the material to fracture at lower stresses [21]. In addition, large pores may also affect the fatigue life and durability of the material.

Number of pores: An increase in the number of pores also reduces the tensile strength of a material. The presence of multiple pores can lead to more stress concentration points, thus reducing the overall mechanical properties of the material.

### 4.3. Strategies to Reduce Porosity

Optimization of process parameters: By adjusting process parameters such as laser energy density and scanning speed, the formation of porosity during LPBF can be reduced. For example, reducing the laser energy density can reduce the formation of keyhole porosity. Use of high quality powder: The quality of the powder has a great influence on the formation of pores during LPBF. Using powders with a uniform particle size distribution and high purity can reduce the formation of pores. Heat treatment: Heat treatment can improve the microstructure of LPBF-forming materials, thereby reducing the number and size of porosity. For example, annealing treatments can eliminate or reduce residual stresses and porosity in the material.

### 4.4. Prospect

The size and number of pores in LPBF forming materials have a significant impact on the tensile strength of the material. By optimizing process parameters, using high-quality powders and heat treatment, the formation of porosity can be effectively reduced and the mechanical properties of the material can be improved. Future research can further explore the mechanism of porosity formation, develop more effective methods to reduce porosity, and design new high-performance alloys that are more suitable for the LPBF process [22].

## 5. Conclusions

This study conducted experimental and simulation analyses on LPBF-formed WE43 magnesium alloy, and the following conclusions were obtained:

- CT inspection revealed the presence of numerous void defects with equivalent diameters ranging from 10∼122 μm in the gauge section of LPBF-formed WE43 magnesium alloy specimens. Approximately 80% of the defects had diameters concentrated in the range of 10∼40 μm, with 56.2% of defects having sphericity values between 0.65∼0.7 μm.
- Low-magnification inspection showed that LPBF-formed WE43 magnesium alloy specimens exhibited various irregularly shaped void defects, with many individual defects below 50 μm in size. These defects included a few fully spherical voids around 20 μm in diameter and unfusioned WE43 powder particles.
- Fitting analysis of the LPBF-formed WE43 magnesium alloy fusion pool revealed an ellipsoidal shape approximately 132 μm long and 96 μm wide, closely resembling actual fusion pool dimensions. Analysis indicated that with a powder layer thickness of 20 μm, each layer of WE43 magnesium alloy powder underwent at least three refusion processes.
- LPBF-formed WE43 magnesium alloy exhibited an ultimate tensile strength of 313 ± 5 MPa, a yield strength of 236 ± 12 MPa, and an elongation of 7.6 ± 0.5%. The microstructure mainly consisted of fine equiaxed grains and columnar grains distributed in a striped pattern. The presence of “scales” significantly influenced grain size and distribution.

## Figures and Tables

**Table 1:** Chemical composition of WE43 powder wt (%).

Zn	Zr	Gb	Nd	Y	Mg
0.21	0.4	1.23	2.46	3.77	Bal.

**Table 2:** Process parameters.

Parameters	Values
Laser Power/W	80
Laser Scanning Speed/mm·s^−1^	800
Powder Layer Thickness/mm	0.02
Scan Spacing/mm	0.07
Scan Vector Angle/°	67
Scan Strategy	Stripe Scanning
Diameter of the focused beam/μm	70

## Data Availability

The data presented in this study are available on request from the corresponding author.
